# Effect of Effluent Recirculation on Biogas Production Using Two-Stage Anaerobic Digestion of Citrus Waste

**DOI:** 10.3390/molecules23123380

**Published:** 2018-12-19

**Authors:** Lukitawesa, Rachma Wikandari, Ria Millati, Mohammad J. Taherzadeh, Claes Niklasson

**Affiliations:** 1 Swedish Center for Resource Recovery, University of Borås, 50190 Borås, Sweden; Mohammad.taherzadeh@hb.se; 2 Department of Food and Agricultural Product Technology, Universitas Gadjah Mada, Yogyakarta 55281, Indonesia; rachma_wikandari@mail.ugm.ac.id (R.W.); ria_millati@ugm.ac.id (R.M.); 3 Department of Chemical Reaction Engineering, Chalmers University of Technology, 41296 Göteborg, Sweden; claesn@chalmers.se

**Keywords:** biogas, anaerobic digestion, citrus waste, recirculation, STR, UASB

## Abstract

Citrus waste is a promising potential feedstock for anaerobic digestion, yet the presence of inhibitors such as d-limonene is known to limit the process. Effluent recirculation has been proven to increase methane yield in a semi-continuous process for recalcitrant material, but it has never been applied to toxic materials. This study was aimed to investigate the effect of recirculation on biogas production from citrus waste as toxic feedstock in two-stage anaerobic digestion. The first digestion was carried out in a stirred tank reactor (STR). The effluent from the first-stage was filtered using a rotary drum filter to separate the solid and the liquid phase. The solid phase, rich in hydrophobic D-limonene, was discarded, and the liquid phase containing less D-limonene was fed into the second digester in an up-flow anaerobic sludge bed (UASB) reactor. A high organic loading rate (OLR 5 g VS/(L·day)) of citrus waste was fed into the first-stage reactor every day. The effluent of the first-stage was then fed into the second-stage reactor. This experiment was run for 120 days. A reactor configuration without recirculation was used as control. The result shows that the reactor with effluent recirculation produced a higher methane yield (160–203 NmL/g·VS) compared to that without recirculation (66–113 NmL/g·VS). More stable performance was also observed in the reactor with recirculation as shown by the pH of 5–6, while without recirculation the pH dropped to the range of 3.7–4.7. The VS reduction for the reactor with recirculation was 33–35% higher than that of the control without recirculation. Recirculation might affect the hydrolysis-acidogenesis process by regulating pH in the first-stage and removing most of the D-limonene content from the substrate through filtration.

## 1. Introduction

Citrus fruits are among the most consumed types of fruit globally due to their massive productivity, low price, and healthy dietary properties [1,2]. Its production throughout the world increased by 20.6% from 2006 to 2016, to 132.4 million tones [3]. Citrus fruits are commonly used for juice production, in which 50% in weight of citrus fruits end up as solid waste during the extraction process [1,2]. The citrus waste has high water (80–90%) and organic matter (95%) content [4]. These properties make citrus waste a potential substrate for anaerobic digestion. However, the low pH (3–4) and the presence of citrus essential oil pose obstacles for this use.

The major component of citrus essential oil (CEO) in citrus waste is d-limonene, which makes up 68–98% of sweet orange essential oil [5]. Several studies have shown the inhibition effect of d-limonene on anaerobic digestion of citrus waste. Mizuki et al. [6] reported that the limit dosage of d-limonene was 26 mg/(L·day). Wikandari et al. [7] reported that the methane production of single-stage anaerobic digestion halted at an organic loading rate (OLR) of 1.5 g VS/(L·day). Their study showed that VFA (volatile fatty acids) started to be accumulated around 7.5 g/L, which indicated that the inhibition occurred in the system at this OLR.

Co-digestion has been studied as a strategy for overcoming the inhibition effect caused by d-limonene [8]. However, the results were not as expected. Co-digestion of citrus waste with cow dung at a ratio of 1:1 resulted in low methane yield [9]. Forgacs et al. [10] previously reported that co-digestion of citrus waste and the organic fraction of municipal solid waste with a percent ratio of 30:70 was successfully conducted up to an OLR of 3 g VS/(L·day). However, the system failed at higher OLRs. Similarly, Martín et al. [11] reported that anaerobic co-digestion of orange peel with glycerol (1:1) resulted in stable operation up to an OLR of 1.91 g VS/(L·day), but failed at higher OLRs. These studies imply that co-digestion of citrus waste is not suitable for operation at high OLRs. In addition, co-digestion can only treat a relatively low amount of citrus waste, which makes it undesirable from an industrial point of view. Hence, an alternative solution is needed.

Two-stage digestion with recirculation is a potential approach to address the inhibition problem. In two-stage digestion, the acid and methane forming processes are separated. Acid forming (hydrolytic, acidogenic, and acetogenic microorganisms) and methane forming (methanogens) microorganisms have different pH requirements for their optimum growth. Methanogens are considered as the microorganisms more sensitive to inhibitors (in this case d-limonene) and low pH. Therefore, the separation of the acid forming from the methane forming process could protect methanogens from an acidic environment. The first-stage has an optimum pH for acidophilic microorganisms 5.0–6.5 [12], whereas the optimum pH for hydrogen production is 5.5–6 [13]. The second-stage, which is dedicated to methane production, has an optimum pH of 7–8 [14]. The accumulation of VFA produced during acidogenesis in the first-stage generally decreases the pH below the optimal value [15].

Another way to protect the methanogens from d-Limonene could be to filtrate the effluent from the acid forming process in the first-stage prior to feeding it into the methane forming process in the second-stage. The d-limonene released from the peel during hydrolysis has low solubility in water [16], and therefore mostly stays with the undigested solid residue.

Anaerobic digestion of citrus waste at high OLRs resulted in a pH drop in the first-stage reactor, which is not favorable for the growth of acid-forming bacteria. In order to maintain the pH, a base can be added to the first-stage reactor. This approach, however, is costly and less environmentally friendly. Gottardo et al. [17] reported that effluent from the second-stage digester is rich in buffering agents. Hence recirculation of the effluent from the reactor of the second-stage might help to maintain the pH in the reactor of the first-stage. Recirculation of effluent from the second-stage into the first-stage in anaerobic digestion of starch and cotton resulted in higher degradability (91% and 80%, respectively) compared to without recirculation (82% and 56%) [18]. Therefore, the aim of this work was to investigate the efficacy of combining two-stage digestion, filtration, and recirculation for mitigating the inhibition effect of the natural low pH and d-limonene content of citrus waste in anaerobic digestion.

## 2. Materials and Methods

### 2.1. Inoculum

The STR (stirred tank reactor) used in the first-stage was inoculated with sludge obtained from the 3000 m^3^ thermophilic biogas plant (55 °C) at Borås Energy and Environment AB (Borås, Sweden). The sludge was stored in an incubator at a temperature of 55 °C for 2–3 days. The sludge was shaken in order to obtain homogenous inoculum. Any remaining large particles were separated by passing the sludge through a sieve with a pore size of 1 mm. An up-flow anaerobic sludge blanket (UASB) reactor used in the second-stage digestion was inoculated with granulated anaerobic sludge, which was obtained from a pilot scale UASB reactor treating municipal wastewater at Hammarby Sjöstad (Stockholm, Sweden).

### 2.2. Citrus Waste

The citrus waste used in this work was orange residue obtained from juice processing at the Brämhult factory (Borås, Sweden) and stored at −20 °C prior to use. The citrus waste was thawed and homogenized to obtain the citrus waste slurry. The characteristic of citrus waste used in this experiment are shown in Table 1. The d-limonene content of the citrus waste was analyzed using gas chromatography–mass spectrometry (GC-MS, Trace GC Ultra, ThemoScientific, Waltham, MA, USA).

### 2.3. Reactors

The reactor used in the first-stage was an STR (Bioprocess Control AB, Lund, Sweden) with a volume of 2 L and a working volume of 1.5 L. The reactor used in the second-stage was a UASB (Scotch Duran, Mainz, Germany) with a volume 0.5 L and a working volume of 0.4 L. The temperature of the first-stage reactor was maintained at 55 °C, while the second-stage reactor was maintained at 37 °C. Both reactors were equipped with a feeding inlet, a liquid sampling point, an effluent outlet, and a gas line to the gas measuring system which was equipped with a gas sampling port.

### 2.4. Experimental Procedure

**Figure 1:**
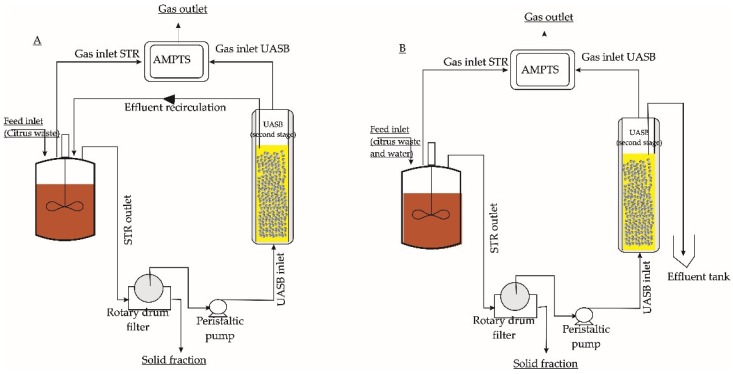
Schematic figure of the semi-continuous two-stage system with liquid recirculation (**A**) and without recirculation (**B**) (modified from Aslanzadeh et al. [20]). AMPTS = Automatic Methane Potential Testing System; STR = Stirred tank Reactor; UASB = up-flow anaerobic sludge blanket.

The STR was seeded with thermophilic anaerobic digestion sludge from biogas plant as mentioned in Section 2.1. The UASB reactor was seeded with granulated anaerobic sludge as mentioned in Section 2.1. The first-stage reactor was fed with citrus waste at OLR of 0.5 g VS/(L·day) and then increased stepwise every 1 week until reaching OLR 5 g VS/(L·day). The two-stage digestion with (Figure 1A) and without (Figure 1B) effluent recirculation. The first-stage of the systems were STR bioreactors with 2 L volume while the second-stage was a up-flow anaerobic sludge blanket (UASB) bioreactor with 0.4 L volume containing flocculated microorganism. The slurry outlet of the first-stage passed a rotary drum filter (Steadfast Equipment, Howell, NJ, USA) containing a polyamide cartridge with pore size of 70 µm to separate liquid from remaining solid particles. The solid particles (retentate) was discharged from filter, while the liquid part was fed into the UASB (second-stage) bioreactors. Both systems were fed with citrus wastes. The differences between the two systems was whether the effluent of the second-stage was fed back to the first stage bioreactor or not (conferatur Figure 1).

For the system with effluent recycling (Figure 1A), the hydraulic retention time (HRT) in the first-stage reactor was 15 days. Prior to feeding, the citrus waste was diluted with effluent from second-stage reactor until the volume reached 100 mL. At the beginning of the experiment (day 0), the OLR was started at 1 g VS/(L·day) and then increased step wise into OLR of 5 g VS/(L·day) during day 0 until day 47. The digestate from first-stage was filtered using rotary drum filter. In the second-stage reactor, the HRT was set at 3 days to adapt the microorganisms. Afterwards, it was shortened step wise into 1.5 days by increasing the volume of the feeding. The whole experiment was carried out in 120 days. The two-stage digestion without recirculation (Figure 1B) was carried out as the same procedure including the HRT, OLR, and filtration. The difference was the liquid that used for diluting the citrus waste was water (not the effluent from second-stage). The liquid from the second-stage was discharged from the system. The whole experiment was carried out in 120 days.

The volume of biogas production was recorded continuously by an Automatic Methane Potential Testing System (AMPTS, Bioprocess Control AB, Lund, Sweden). The biogas composition was analyzed using gas chromatography. The liquid and gas sampling were performed 3–4 times a week. The liquid samples were kept at −20 °C until the analyses were performed.

### 2.5. Analytical Method

#### 2.5.1. Analysis of Gas Fraction

The composition of biogas production was measured using a gas chromatograph (Auto System Perkin-Elmer, Waltham, MA, USA), equipped with a packed column (Perkin Elmer, 6′ × 1.8′′ OD, 80/100 Mesh) and a thermal conductivity detector (Perkin Elmer). The injector temperature was 150 °C, detection temperature was 200 °C, and oven temperature was 75 °C. The carrier gas was pure nitrogen at a pressure of 0.70 bar and a flow rate of 40 mL/min at 60 °C. A 250 µL pressure-tight gas syringe (VICI, Precision Sampling Inc., Baton Rouge, LA, USA) was used for the gas sampling.

#### 2.5.2. Analysis of Liquid Fraction

Liquid samples were analysed for pH, soluble COD, and VFA concentration after centrifugation at 17,000 × *g* for 10 min and subsequent filtration through a 0.2-µm filter to remove solid particles. Glucose and fructose content of substrate were determined with Sucrose/d-Fructose/d-Glucose assay kit (Megazyme, Chicago, IL, USA). The COD was measured using a HACH apparatus equipped with a UV–Vis spectrophotometer (HACH, Düsseldorf, Germany). Digestion solution COD-kit (HACH, NANOCOLOR^®^ Düsseldorf, Germany) with a detection range of 1–15,000 g/L was used. The concentration of VFAs including acetic, propionic, butyric, isobutyric, valeric and isovaleric acid were analyzed by HPLC (Waters 2695, Waters Corporation, Milford, MA, USA), which was equipped with an ion-exchange column (Aminex HPX-87H Bio-Rad, Hercules, CA, USA) and a Waters 2414 UV detector. The column temperature was set at 60 °C and 5 mM sulfuric acid was used as eluent with a flow rate of 0.6 mL/min.

Analysis of d-limonene in the substrate, eluent and solid retentate were conducted using gas chromatography coupled to a mass spectroscopy detector (GC-MS Trace GC Ultra, ThemoScientific, Waltham, MA, USA). The GC-MS was equipped with silica capillary column (ZB-5MS fused-silica with 30 m × 0.25 mm id × 0.25 μm film). Helium was used as the carrier gas with a flow rate of 1.2 mL/min. The temperature of the column was set at 50 °C for 2 min and the temperature was increased to 120 °C with a rate of 4 °C/min. The injector temperature was set at 250 °C, and the detector temperature was set at 280 °C. Before analysis, the citrus essential oil was extracted from the sample using Soxhlet method with minor modification [21,22]. Ten grams of material was diluted with 200 mL of heptane. The extraction was run for three hours and the solvent was removed by evaporation to achieve 50 mL solvent extract. The solvent extract was filtered through a 0.2 μL syringe filter before injected to GC-MS. Nonanal was added as an internal standard.

#### 2.5.3. Analysis of Solid Fraction

TS (total solid) and VS (volatile solid) of inoculum and substrate were analyzed with the thermogravimetric method, according to the laboratory analytical procedure by Sluiter et al. [23].

### 2.6. Calculation

The methane can be calculated theoretically using the following equation [24]:

C_c_H_h_O_o_N_n_S_s_ + yH_2_O → xCH_4_ + nNH_3_ + sH_2_S + (c − x) CO_2_ (1)

Mol of methane produced (x) = 1/8(4c + h − 2o − 3n − 2s)

The digestibility of citrus waste calculated as:

(2) $$\frac{Experimental\;methane\;production}{Theoretical\;methane\;production}\times 100\%$$

## 3. Results and Discussion

The first report showing that d-limonene could inhibit methane-forming microorganisms in the rumen was published in 1957 [25]. Since then, several studies have attempted to digest citrus waste in different ways. However, most of the digestion halted at maximum OLR of 2 g VS/(L·day) [6,26,27,28]. In this stud, several approaches combining two-stage digestions, filtrations, and an effluent recirculation have been investigated in order to increase the methane production. The first-stage was carried out in a STR whereas the second-stage was carried out in an UASB reactor. In the system with recirculation, the effluent from the second-stage was transferred to the first-stage. In order to evaluate the effect of recirculation, several parameters were measured, including the VS reduction and pH in the first-stage, as well as the biogas production and composition in the second-stage reactor. Two-stage anaerobic digestion of citrus waste in a system without recirculation was also conducted as control.

### 3.1. Effect of Effluent Recirculation on Acidogenesis of Anaerobic Digestion of Citrus Waste In the First-Stage

**Figure 2:**
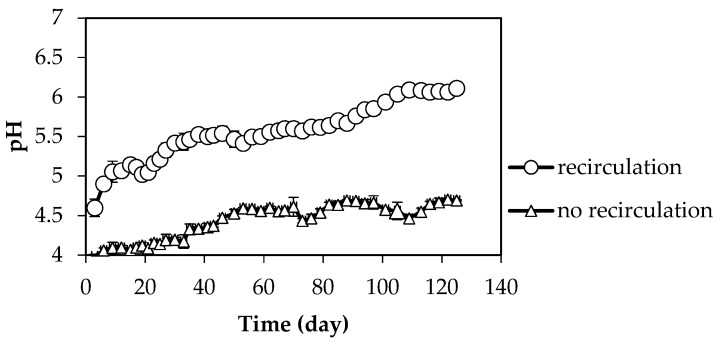
The changes of pH of the first-stage reactor in the system with and without recirculation.

The effect of recirculation on pH during the first-stage of anaerobic digestion of citrus waste is presented in Figure 2. The pH in the first-stage reactor with recirculation increased gradually from 4.5 to 6.1, whereas the pH in the reactor without recirculation only increased from 4.4 to 4.7. This might be explained by high amounts of buffering agents such as bicarbonate and ammonia [29] in the effluent from the second-stage, which could aid in controlling the pH in the first-stage [29,30,31]. The effluent from the second-stage in reactor with recirculation has a pH in the range of 7–7.9 (data not shown). This phenomenon was also observed by Aslanzadeh et al. [18] in anaerobic digestion of cotton and starch, in which the pH of effluent from the second-stage was 8. The recirculation of the effluent caused the pH of the first-stage to stabilize over 6.

The higher pH in the reactor with recirculation leads to a higher production of VFAs (Table 2). Volatile fatty acids (or short chain fatty acids) have a pKa of around 4.8 [32]. VFAs can be toxic to microorganisms, and their toxicity depends on the pH. In the first-stage in the reactor without recirculation, the pH was lower than the pKa. When the pH in the reactor is lower than its pKa, the VFAs remain in undissociated form. The undissociated forms of VFAs are hydrophobic compounds. This allows them to pass through cell membranes consisting of a phospholipid bilayer, and to disrupt cell systems [33]. Hence, the production of VFAs is reduced. This would explain why there are higher levels of VFAs in the first-stage with recirculation.

Besides having a negative impact on the first-stage, low pH also affects methane producing microorganisms in the second-stage, as methanogens exhibit optimum growth at pH of 6.7–7.5 [24]. The lack of l-muramic acid in the cell membrane structure of methanogens makes them more sensitive to acids. The impact of a low pH is more pronounced in a one-stage system where acidification and methanogenesis occur in the same reactor. Since a low pH increases the antimicrobial activity of essential oil [34], the presence of d-limonene in the reactor combined with a low pH enhances its toxic effect. In order to control pH levels in the first-stage where acidification occurs, a base solution can also be added to the reactor. However, this approach is undesirable due to the economic cost and environmental impact of the production of this chemical. Hence, recirculation offers an advantage as no additional chemical is needed.

**Figure 3:**
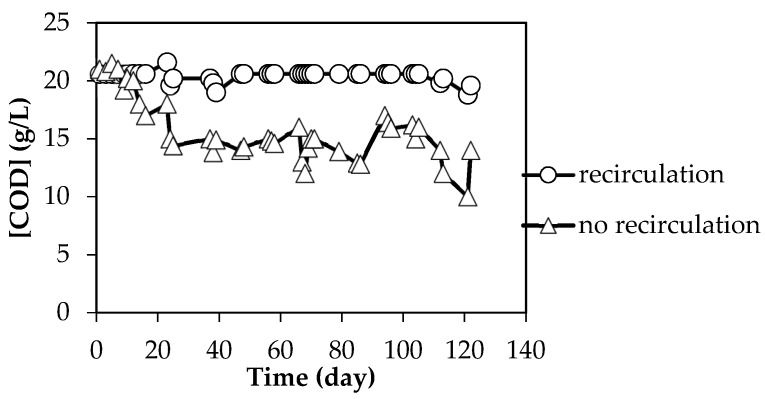
The profile of COD of filtrate from first-stage reactor (STR) with and without recirculation in the system.

Recirculation not only positively affected the pH and VFA production, but also improved substrate degradation. This was indicated by a higher and more stable soluble COD value in the filtrate compared to the reactor without recirculation (Figure 3). The higher VS reduction in the recirculation reactor is in accordance with the higher VFA production. VS reduction of the system with recirculation was 32–34%, while for the system without recirculation it was 15–21%. One possible explanation could be that the reactor with recirculation has an optimum pH for VFA production. In addition, the performance improvement with recirculation might be related to the shifting of the microbial community towards the acidogenic and acetogenic community as suggested by Giulano et al. [15]. With the approximate values of 33% and 18% in the reactors with and without recirculation, respectively, the VS reduction is comparatively low. The reason for this could be that the OLR used in this study was high at 5 g VS/(L·day), as was the loading rate of d-Limonene. Nonetheless, if recirculation is applied to a non-toxic substrate such as starch, the degradability can reach up to 91% of the theoretical value [18].

### 3.2. Effect of Effluent Recirculation on Methane Production of Anaerobic Digestion of Citrus Waste in the Second-Stage

An up-flow anaerobic sludge blanket (UASB) reactor was used as the second-stage reactor in this study because this type of reactor is considered more effective and shows higher resistance to toxicity than a stirred tank reactor (STR) configuration [27]. The VFA-rich filtrate of effluent from the first-stage was fed into the second-stage to be further converted into methane. The HRT of both the recirculated and non-recirculated system was shortened from 3 to 1.5 days to help the microorganisms adapt.

The pH of the effluent from the reactors in the second-stage in both systems was stable above 7, indicating that both reactors were able to consume most of the VFA. This is also shown by the low VFA concentration in the effluent that is correlated to 90% of VFA consumption in both systems (Table 3). In addition, the higher pH above 7 also indicates that buffer agents are present in the effluent from the reactors in the second-stage. In line with VFA consumption, the COD was reduced by 90% in the second-stage reactor for both systems (Table 3). The high COD reduction in the second-stage could be explained by the lower d-limonene content in the filtrate (0.2%) compared to the solid phase (0.8%). d-Limonene is insoluble in water; hence it tends to be retained in the solid phase rather than in the liquid phase. These results prove the advantage of filtration for D-limonene removal in two-stage digestion.

**Figure 4:**
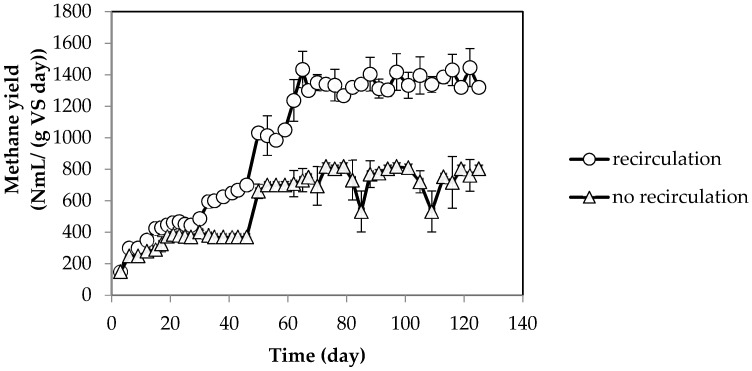
The methane production of second-stage reactor (UASB) with and without recirculation in the system.

Biogas composition in both systems with and without recirculation was similar (Table 3). The methane content in both systems was within a similar range as other reports [35,36,37]. No hydrogen was detected in either system. These results reveal that recirculation did not affect biogas composition. The methane yield from the second-stage is shown in Figure 4. The results showed that methane production was two times higher in the system with recirculation (160–203 NmL/(g·VS·day)) compared to the system without recirculation (66–113 NmL/(g·VS·day)). The higher VFA in the effluent of the first-stage in the system with recirculation might be the reason for the higher methane production. Similarly, Stabnikova et al. [38] reported that 40% higher methane production was obtained by recirculation of effluent from the second-stage into the first-stage in anaerobic digestion of food waste. The authors proposed that recirculation could enhance the acidogenesis process by preventing an excess of acid and providing a faster supply of nutrients to the microorganism. A significant effect of recirculation was observed in two-stage anaerobic digestion of vegetable waste at high OLR (above 2.6 g·VS/(L·day)) [39].

Few studies have attempted to find out the mechanism behind the improvement of the anaerobic digestion performance by recirculation. Degueurce et al [40] reported that effluent recirculation from anaerobic digestion of manure produced biotic and abiotic effects. The biotic effect is changing the microbiological community, whereas the abiotic effect is providing nutrients, adjusting pH and buffering the system. Hence, anaerobic digestion with biotic effluent recirculation produced 50% more methane compared to anaerobic digestion with abiotic effluent recirculation [40]. Recirculation increased the proportion of phylum *Firmicutes* that has two-fold cellulolytic capabilities, which caused better cellulose degradation and higher VFA production [41]. Higher VFA concentration promotes the shift of methanogen genera from *Methanosarcina* spp. to *Methanothermobacter* spp. which is more tolerant to a high concentration of VFA [41]. Digestate recirculation has been also applied in pilot scale and the results proved that recirculation could stabilize the first-stage process [17].

The digestibility of citrus waste in the system with effluent recirculation was 25–33%. The OLR used in this study (5 g·VS/L/day) was relatively high compared to other previous works. Forgacs et al. [10] investigated methane production from co-digestion of untreated citrus waste and the organic fraction of municipal solid waste with a ratio of 30:70 at OLR 3 g VS/L/day and the process ceased after 26 days. Flotats et al. [8] also investigated co-digestion of citrus waste (50–80% *w/w*) and manure with a maximum OLR of 1 g VS/L/day. Wikandari et al. [7,42] studied the resilience of membrane bioreactors against d-Limonene in a synthetic medium and in real citrus waste. However, the OLR of the citrus waste was only investigated until an OLR of 3 g VS/L/day. Reverse membrane bioreactors have been of interest for the biorefinery process of inhibitor containing substrates [43]. However, the high price and intensive maintenance requirements limit their application in industry. Hence, recirculation, which plays the role of pH regulator in the acidification stage, is simpler compared to membrane technology, making it more applicable in industry. The result of the current work reveals that the combination of two-stage digestion, filtration, and effluent recirculation can be successfully applied to anaerobic digestion of citrus waste at high OLRs. However, further studies such as microorganism’s community analysis might be performed in order to broaden the knowledge in this study.

## 4. Conclusions

Recirculation has been shown to successfully increase the methane yield of anaerobic digestion of citrus waste in a two-stage process. Furthermore, recirculation system gives higher degradability as shown by VS reduction and VFA content than that of without recirculation system. Having recirculated the second-stage effluent into the first-stage reactor helped to increase the pH in the first-stage reactor. This leads a better condition for VFA production in the first-stage. In addition, filtration can be used to minimize the amount of D-limonene entering the system in the second-stage. Based on these results, it can be concluded that the combination of recirculation and filtration can be a promising strategy for anaerobic digestion of citrus waste at high OLRs.

## Figures and Tables

**Table 1:** Characteristics of the citrus waste used in this experiment [19].

Components	Content (% db *)
Total Solid (TS)	23.99
Volatile Solid (VS)	23.07
Water	79.07
Ash	0.88
Fat	0.23
Total Protein	1.28
Crude Fiber	3.28
Carbohydrate	18.54
Starch	0.62

* db = dry basis.

**Table 2:** VFA composition and sugar content of filtrate from the first-stage reactor with and without recirculation.

The Soluble Content of the Filtrate	The First-Stage Reactor with Recirculation (g/L)	The First-Stage Reactor without Recirculation (g/L)
	Day 47–125	Day 0–125
Acetate	7.9 ± 0.9	2.5 ± 0.8
Propionate	0.8 ± 0.2	1.1 ± 0.6
Isobutyrate	0.1 ± 0.0	0.3 ± 0.1
Butyrate	0.1 ± 0.0	0.8 ± 0.1
Isovalerate	0.0 ± 0.0	0.0 ± 0.0
Valerate	0.2 ± 0.0	0.0 ± 0.0
Caproate	0.3 ± 0.0	0.0 ± 0.0
Sugar (glucose and fructose)	9.7 ± 0.9	8.2 ± 1.0

**Table 3:** pH, total VFA, COD reduction of effluent from the second-stage reactor with and without recirculation.

Parameter	The Second-Stage Reactor with Recirculation Day 47–125	The Second-Stage Reactor without Recirculation Day 47–125
pH	7.9–8.2	7.8–8.2
Total VFA concentration in the effluent (g/L)	0.8–0.9	0.4–0.45
COD reduction (%)	89–91	90–92
CH_4_ (%)	61–73	60–74
CO_2_ (%)	28–389	256–40
